# Evaluation of Improvement in Skin Laxity and Oiliness After Monopolar Radiofrequency Treatment: A Pilot Study Utilizing Multiple Assessment Tools

**DOI:** 10.1111/jocd.70463

**Published:** 2025-09-27

**Authors:** Dong Hye Suh, Sang Jun Lee, Kye Yong Song, Haerin Yang, Su Ji Chae, Hwa Jung Ryu

**Affiliations:** ^1^ Arumdaun Nara Dermatologic Clinic Seoul Korea; ^2^ Department of Pathology, College of Medicine Chung‐Ang University Seoul Korea; ^3^ Department of Dermatology Korea University Ansan Hospital Ansan Korea

**Keywords:** collagen, elasticity, elastin, histological review, laxity, monopolar radiofrequency, multiparameter skin analysis unit, sebum, skin rejuvenation, three‐dimensional skin analysis device

## Abstract

**Background:**

Radiofrequency (RF) therapy is a popular noninvasive skin‐tightening method, yet objective evaluations of its effectiveness remain limited.

**Aims:**

This study assesses RF therapy's impact on facial skin laxity and oiliness using a 3D skin analyzer, multi‐parameter skin analysis, and histological analysis.

**Methods:**

Thirty‐two patients (skin types III–V) underwent full‐face monopolar RF treatment, with follow‐ups over 24 weeks. Clinical photographs, 3D imaging, and specialized probes measured elasticity and sebum levels. Skin biopsies were taken before and 2 months post‐treatment. Improvement was assessed through patient questionnaires and dermatologist evaluations.

**Results:**

Significant improvements in jawline sagging and nasolabial folds were observed in 2D/3D images. Quantitative analysis confirmed RF‐induced skin rejuvenation, with a statistically significant increase in skin radiance at 4 and 8 weeks. Sebum production decreased notably at 8 and 24 weeks, as confirmed by the sebum index. Elasticity improved, with increased viscoelasticity and reduced retraction time. Histological analysis revealed reduced dermal inflammation, decreased sebaceous gland volume, and enhanced collagen and elastic fiber density. At 2 months, 53.1% of patients reported over 50% improvement. No severe adverse events occurred.

**Conclusion:**

Monopolar RF effectively enhances skin laxity by remodeling collagen and reorganizing elastic fibers. Histological findings and skin analyzer data support RF as a reliable, noninvasive treatment for improving skin firmness and reducing oiliness.

## 1 Introduction

Skin, serving as the primary barrier between the organism and the environment, protects against pathogen invasion and withstands chemical and physical assaults. Throughout an individual's lifespan, skin undergoes numerous changes and transitions, resulting in significant differences between young and aged skin [1]. Skin aging, influenced by both the passage of time (intrinsic) and cumulative exposure to external factors (extrinsic), is characterized by distinct clinical signs such as rhytides, fine wrinkles, hyperpigmentation, thinning, laxity, and dryness. These visible alterations correspond with histological changes in aged skin, including a reduced epidermal thickness, loss of rete ridges at the dermo‐epidermal junction, and changes in dermal structures. The most significant microscopic alterations in the dermis with age include a reduction in collagen and elastin fibers, an increase in collagen cross‐linking, and a degradation of proteoglycans [2].

Given the rise in life expectancy and the social emphasis on appearance, concerns about aged skin have become prevalent among adults, thereby increasing demand for skin rejuvenation therapies. Surgical options such as facelifts have been developed to address sagging skin. However, many patients are reluctant to undergo these procedures due to their invasiveness and extensive recovery periods. Consequently, light‐based devices, including intense pulsed light (IPL), pulsed dye lasers, and Nd:YAG lasers, have gained popularity as minimal and noninvasive alternatives [3, 4]. However, their efficacy in skin tightening is somewhat limited, likely due to the scattering of light within the skin, which hinders its reach to deeper layers. In contrast, high‐intensity focused ultrasound (HIFU) and radiofrequency (RF) devices have shown potential in treating sagging skin by delivering thermal or non‐thermal energy directly to the targeted area, promoting tissue coagulation and subsequent remodeling [5, 6].

RF therapy has indeed become widely recognized as an effective, noninvasive option for skin rejuvenation and tightening. The electromagnetic field generated by radiofrequency stimulation alters the polarity of particles in the skin tissue approximately 6 million times per second. These rapid movements of charged particles generate high‐frequency electrical currents [6]. The natural resistance of the skin tissue converts these electrical currents into volumetric heating, causing controlled damage to collagen fibrils. This process induces a tightening effect through the immediate contraction of collagen fibrils, followed by the stimulation of new collagen synthesis [7].

To the best of our knowledge, there have been limited trials aimed at objectively assessing the efficacy of monopolar RF on improving facial skin laxity from diverse perspectives. Earlier studies typically assessed its effectiveness through subjective questionnaires or using a singular measurement tool. Therefore, this study seeks to comprehensively validate the hypothesis that monopolar RF enhances skin rejuvenation and tightening by utilizing multiple assessment modalities that provide both quantitative and objective data.

## 2 Materials and Methods

### 2.1 Patients

Thirty‐two patients (thirty females and two males) with moderate to severe skin laxity participated in this study, which was conducted at the Department of Dermatology, Korea University Ansan Hospital, and Arumdaun Nara Clinic, South Korea. The study protocol was approved by the Institutional Review Board of Korea University Ansan Hospital (IRB no. 2023AS0243). Their demographic data are shown in Table 2. Prior to undergoing the procedure, all participants were fully informed of the potential benefits and risks associated with the treatment, and written informed consent was obtained from each patient.

### 2.2 Inclusion and Exclusion Criteria

This study involved healthy adults aged 25–80 with Fitzpatrick skin types III to V who sought skin tightening. Patients with a keloid history, active cutaneous infections, photosensitivity, uncontrolled general diseases, or electric device implants such as pacemakers were excluded from participation. Additionally, individuals who had undergone laser treatments, chemical peels, or facial plastic surgery within the past 6 months, as well as those who were pregnant or lactating, were also excluded.

### 2.3 Study Design and Clinical Assessment

All patients received a single session of monopolar RF (Density, Jeisys Medical Inc., Seoul, Republic of Korea) and were monitored at 4, 8, and 24 weeks post‐treatment. A topical anesthetic cream (eutectic mixture of 2.5% lidocaine HCl and 2.5% prilocaine; TaiGuk Pharm Co. Ltd.) was applied 40 min prior to treatment for local anesthesia. Treatment intensity levels ranged from 2.0 to 3.5, and the total delivered energy varied from 47.3 to 55.9 kJ/cm^2^ (mean energy 51.70 kJ/cm^2^). Follow‐up visits at 4, 8, and 24 weeks included the capture of clinical photographs and three‐dimensional (3D) images. Digital photographs were taken using a Canon EOS 80D camera, and 3D images were captured with a 3D Meta‐vu camera (PSI Plus, Suwon, Republic of Korea). Clinical photographs were taken with the patient's eyes closed under appropriate lighting conditions. Conversely, 3D photographs were captured with the patient's eyes open or closed, following verbal instructions, using four different flashlight modes: normal, specular, polarized, and ultraviolet light.

Its therapeutic effects were also assessed using the multi‐parameter skin analysis device (DermLab Combo Series Skinlab Combo; Cortex, Denmark). Measurements of viscoelasticity (VE), retraction time (RT), and sebum production were conducted at each visit.

Skin biopsies were conducted on twelve subjects who consented to the invasive procedure. Paraffin‐embedded tissue samples obtained from patients treated with monopolar RF at baseline and 2 months post‐treatment were stained using hematoxylin and eosin (H&E), Masson's trichrome (MT), and Victoria blue (VB) to evaluate histological changes.

Any adverse effects, such as fat atrophy, erythema, edema, pain, and burn, were assessed during the treatment period. At 24 weeks post‐treatment, skin laxity improvement was evaluated through questionnaires or photo reviews by patients and blinded investigators, using quartile scoring as described in Table 1. The grading was conducted by two independent, blinded, board‐certified dermatologists.

**TABLE 1:** Scoring scales to assess overall improvement of skin conditions subjectively and objectively.

Grade	The degree of improvement
1	No improvement
2	1%–24% improvement
3	25%–49% improvement
4	50%–74% improvement
5	75%–100% improvement

### 2.4 Statistical Analysis

Statistical analyses were conducted using SPSS ver. 29. A paired sample *t*‐test evaluated within‐subject differences for variables measured by the multi‐parameter skin analysis device. Repeated‐measures analysis of variance (RM ANOVA) was used to compare data from the 3D skin analyzer at each follow‐up visit. A *p*‐value < 0.05 was regarded as statistically significant.

## 3 Results

Thirty‐two patients (30 women and 2 men) underwent treatment with a monopolar RF device. As outlined in Table 2, the average age of these patients was 51.6 years (range, 36–76 years; median age, 52 years), with Fitzpatrick skin types III, IV, or V. All patients completed one treatment session and follow‐up visits at 4, 8, and 24 weeks post‐treatment.

**TABLE 2:** Demographic data of enrolled patients.

Characteristic	Value
Sex, *n* (%)	
Male	2 (6.25)
Female	30 (93.75)
Age, years	
Range (median)	36–76 (52)
Mean (SD)	51.6 (7.81)
Fitzpatrick skin type, *n* (%)	
III	13 (40.625)
IV	14 (43.75)
V	5 (15.625)

**FIGURE 1:**
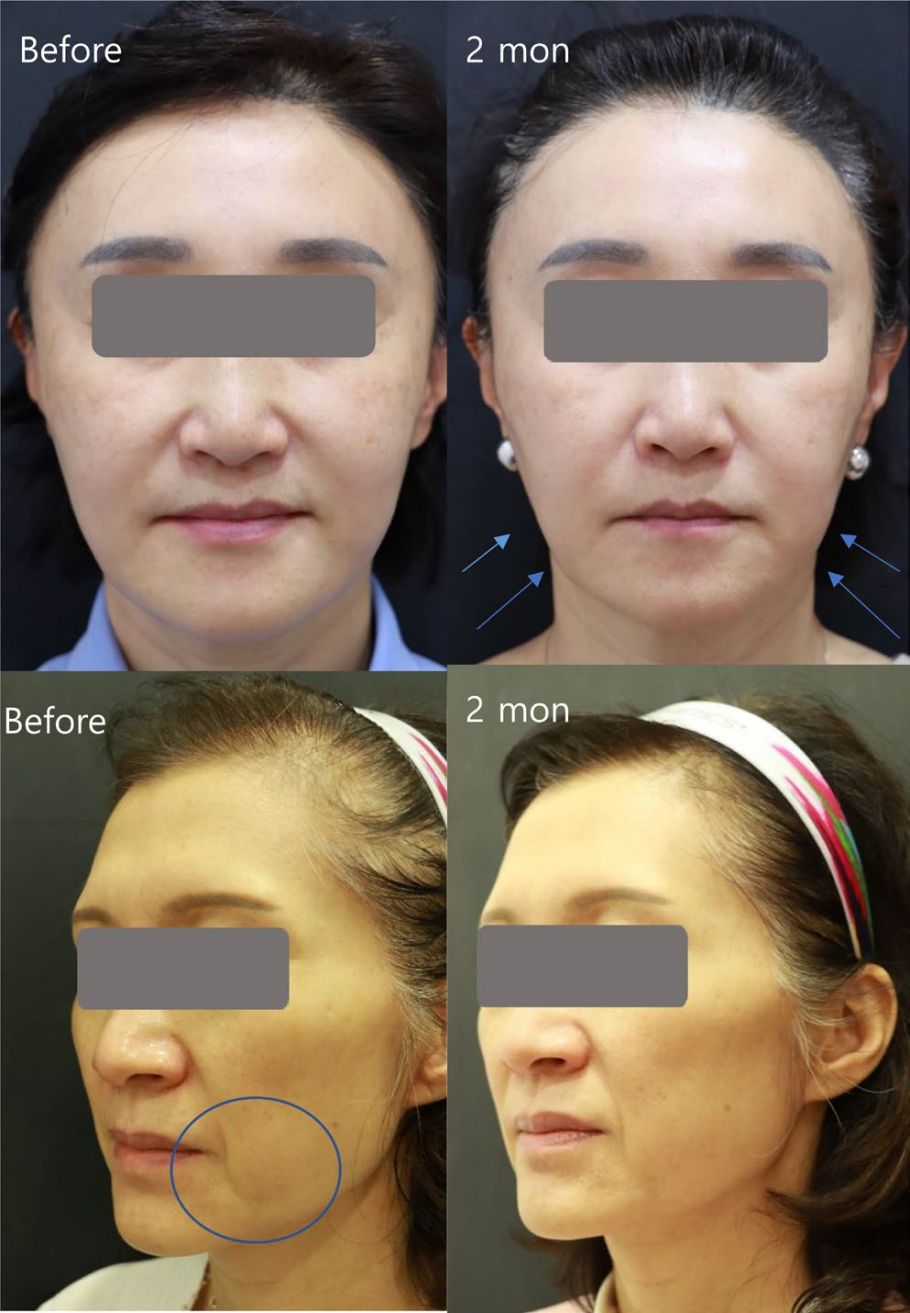
Representative clinical photographs showing improvement of skin laxity along the jawline. A 55‐year‐old female (above) exhibited skin tightening of the jowls and nasolabial fold improvement after undergoing monopolar RF treatment. A 60‐year‐old female (below) exhibited improvement of Marionette line after RF treatment.

**FIGURE 2:**
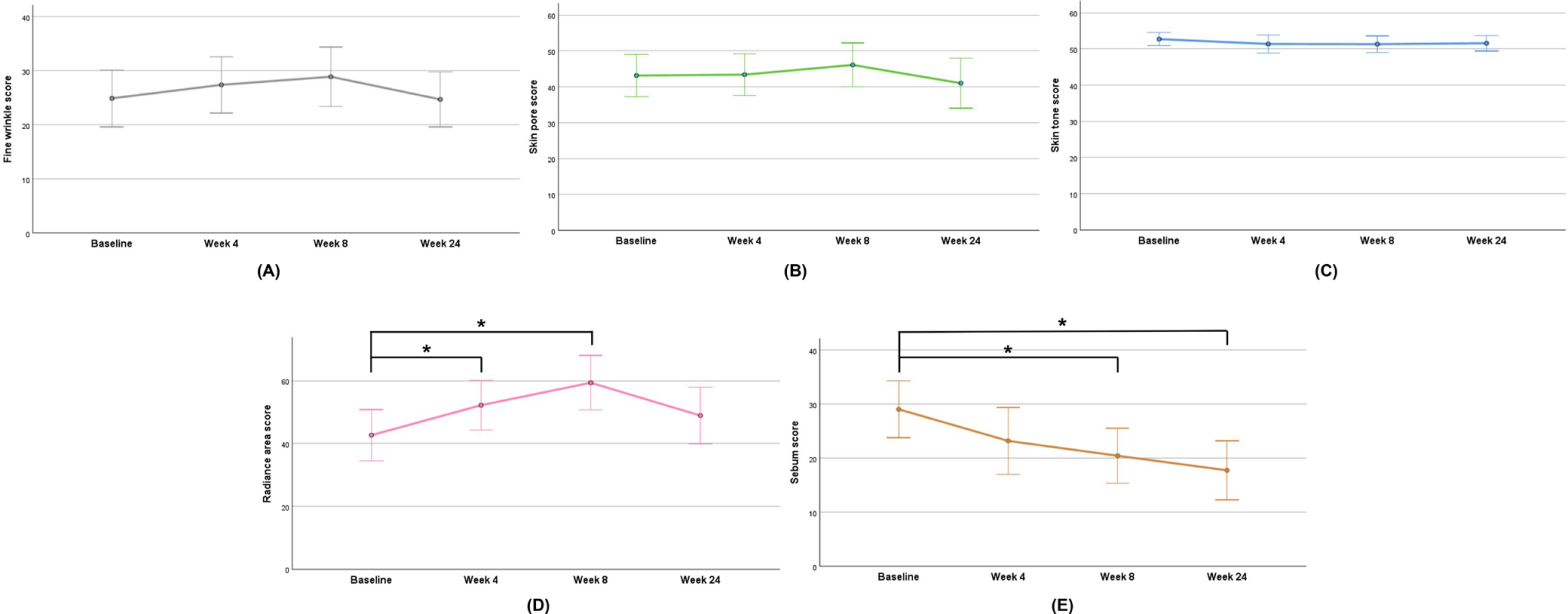
Assessment of fine wrinkles, skin pores, skin tone, radiance, and sebum using 3D skin analyzer device. There were no statistically significant changes in the scores of fine wrinkles (A), skin pores (B), and skin tone (C) over time. However, significant differences were observed in the radiance (D) and sebum (E) scores, prompting post hoc tests to determine the specific groups that differed from the baseline for these variables. These analyses revealed that radiance scores significantly increased at 4 (*p* = 0.037) and 8 weeks (*p* < 0.001), while sebum scores significantly decreased at 8 (*p* = 0.016) and 24 weeks (*p* < 0.001) compared with baseline. Data were analyzed using RM ANOVA. The asterisk indicates a significant difference. *p < 0.05.

Clinical photograph analysis indicated improvements in sagging jawlines and prominent nasolabial folds following the treatment, as shown in Figure 1. The 3D skin analyzer machine verified these skin rejuvenation effects, capturing images that enhanced the visibility of fine wrinkles, pores, sebum, and radiance under different light sources. Interestingly, the analyzer also provided numerical values for each skin index, allowing for statistical analysis of quantitative variables. For instance, the radiance score significantly increased from 42.65 ± 17.48 at baseline to 52.20 ± 16.94 at 4 weeks (*p* = 0.037), reached 59.35 ± 18.60 at 8 weeks (*p* < 0.001), and then decreased to 48.90 ± 19.31 at 24 weeks (*p* = 0.360). The rise in radiance at 4 and 8 weeks post‐treatment suggests an improvement in skin texture. Similarly, skin oiliness improved with significant reductions in the sebum score at 4, 8, and 24 weeks post‐treatment compared to baseline levels. The sebum score decreased from 29.05 ± 11.24 to 23.20 ± 13.24 at 4 weeks (*p* = 0.144), 20.45 ± 10.86 at 8 weeks (*p* = 0.016), and 17.75 ± 11.68 at 24 weeks (*p* < 0.001). However, no statistically significant changes were observed in scores for fine wrinkles, pores, and skin tone throughout the study period, as shown in Table 3 and Figure 2.

**TABLE 3:** Changes in fine wrinkles, skin pores, skin tone, radiance, and sebum scores measured by a 3D skin analyzer device.

	Baseline	Week 4	Week 8	Week 24	*p* (95% CI)
	Mean ± SD	Mean ± SD	Mean ± SD	Mean ± SD	
Fine wrinkle score	24.90 ± 11.29	27.40 ± 11.14	28.90 ± 11.73	24.70 ± 10.85	0.306
Skin pore score	43.15 ± 12.47	43.40 ± 12.45	46.10 ± 13.11	41.00 ± 14.83	0.308
Skin tone score	52.70 ± 3.95	51.35 ± 5.38	51.30 ± 4.93	51.55 ± 4.52	0.186
Radiance score	42.65 ± 17.48	52.20 ± 16.94	59.35 ± 18.60	48.90 ± 19.32	**< 0.001**
Sebum score	29.05 ± 11.24	23.20 ± 13.24	20.45 ± 10.86	17.75 ± 11.68	**< 0.001**

*Note:* Data were analyzed using RM ANOVA. Values deemed statistically significant (*p* < 0.05) are highlighted in bold.

Abbreviations: CI, confidence interval; SD, standard deviation.

Assessment of overall improvement is advised through patients' responses to questionnaires (subjective assessment) and investigators' review of clinical photography (objective assessment). They have used the scoring system in Table 1. According to these assessments, 53.1% and 62.5% of treated patients reported more than 50% improvement at the 2‐month follow‐up, respectively (Table 4).

**TABLE 4:** Overall improvements as assessed by patients and investigators.

Grade of overall improvement	Patients' assessment: *N* (%)	Physicians' assessment: *N* (%)
Grade 1	1 (3.1%)	0 (0%)
Grade 2	7 (21.9%)	3 (9.4%)
Grade 3	7 (21.9%)	9 (28.1%)
Grade 4	9 (28.1%)	15 (46.9%)
Grade 5	8 (25.0%)	5 (15.6%)

Eight cases of non‐severe adverse events were reported during the study. Five of these eight presented with mild to moderate erythema immediately after the treatment, which resolved within a short period with or without topical steroids (Dermatop ointment; Prednicarbate; HanDok Pharm Co. Ltd.). Additionally, three patients experienced small‐sized burns on the day of RF treatment, treated effectively with topical antibiotics (Bearoban ointment; Mupirocin; HanAll Biopharma Ltd.) and hydrogel dressings (DuoDERM Extra Thin; Convatec Group; USA), leading to healing without post‐inflammatory hyperpigmentation (PIH).

### 3.1 Multi‐Parameter Skin Analysis and Histologic Assessment

As determined by the multi‐parameter skin analysis unit, the average sebum production level decreased from 32.88% ± 31.80% to 12.25% ± 20.39% 2 months post‐treatment, a statistically significant reduction according to the paired *t*‐test (*p* = 0.005). Furthermore, viscoelasticity (VE) increased from 4.61 ± 3.00 MPa to 6.06 ± 2.84 MPa, while retraction time decreased from 242.00 ± 172.99 to 167.86 ± 68.16 ms. Both the increase in VE and decrease in RT were statistically significant (VE: *p* = 0.023; RT: *p* = 0.017), evidencing the efficacy of monopolar RF treatment in enhancing skin elasticity (Table 5).

**TABLE 5:** Changes in sebum index, viscoelasticity (VE), and retraction time (RT) measured by a multi‐parametric skin analysis unit before and after treatment.

	Pre‐treatment	Post‐treatment	*p* (95% CI)
	Mean ± SD	Mean ± SD	
Sebum production (%)	32.88 ± 31.80	12.25 ± 20.39	**0.005**
Viscoelasticity (MPa)	4.61 ± 3.00	6.06 ± 2.84	**0.023**
Retraction time (ms)	242.00 ± 172.99	167.86 ± 68.16	**0.017**

*Note:* Two months post‐treatment, sebum production was significantly reduced compared to baseline levels, indicating the oil‐reducing effect of RF on the skin. Additionally, there were statistically significant increases in VE and decreases in RT, suggesting that RF may enhance skin elasticity. Data were analyzed using a paired *t*‐test. Values deemed statistically significant (*p* < 0.05) are highlighted in bold.

Abbreviations: CI, confidence interval; SD, standard deviation.

**FIGURE 3:**
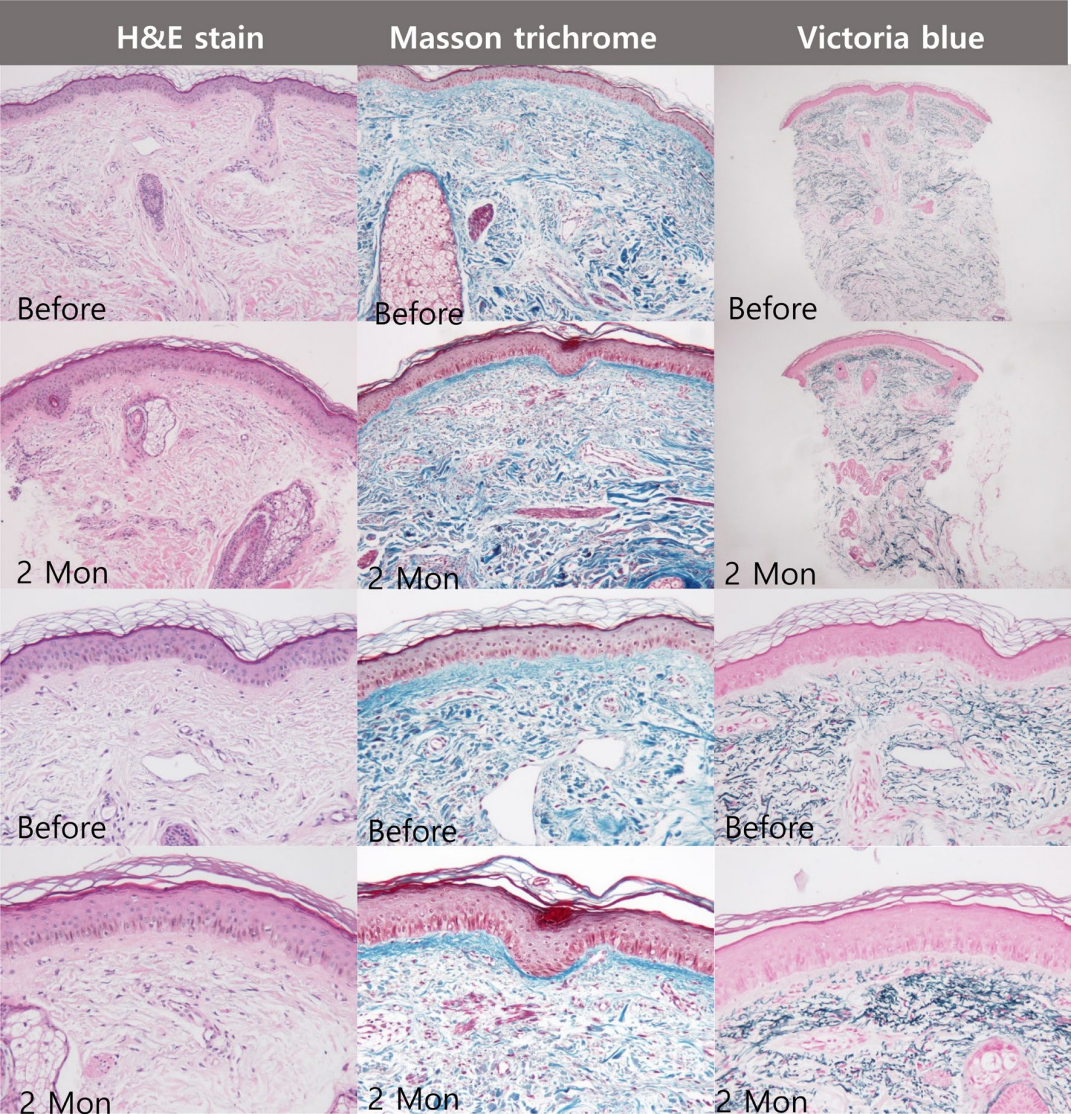
Histological modifications following monopolar RF treatment. Histology slides stained with various methods including H&E, M‐T, and VB stains for two patients who demonstrated significant improvement either in sagging jawline or prominent jowls are displayed. Decreased solar elastosis and increased collagen fibers were noted in the H&E stain. Increases in the density of collagen and elastic fibers were evident in the M‐T and VB stains, respectively.

To assess its effectiveness in skin rejuvenation from a histopathological perspective, paraffin‐embedded tissue samples were collected from 12 subjects who consented to additional invasive procedures at both baseline and 2 months post‐treatment. Punch biopsies with a diameter of 2 mm were used on the anterior cheek. Post‐treatment, the H&E stain demonstrated reduced dermal vessels, reduced solar elastosis, and increased collagen fibers. In Masson's trichrome stain, collagen fiber density significantly improved, showing thickened bands in the papillary dermis. Moreover, Victoria blue stain, used for elastic fibers, showed an increased density of dermal elastic fibers (Figure 3). These results support the hypothesis that monopolar RF devices may tighten sagging skin by enhancing the connective tissue framework and ameliorating histopathological age‐related changes.

## 4 Discussion

The global market for skin rejuvenation devices has expanded significantly over recent decades, driven by the aging population and their desire to maintain a youthful appearance. To meet this demand, a range of sophisticated and innovative treatment options has consistently been introduced to this burgeoning market [8]. Nonablative monopolar RF treatment has become popular due to its minimally invasive nature and minimal downtime, allowing patients to continue with their daily activities uninterrupted [9]. Monopolar RF treatment is regarded as an effective modality for skin rejuvenation, facilitating facial contouring and tightening through volumetric heating and collagen remodeling in the dermis.

The monopolar RF device consists of three components: a generator, a cryogen unit, and a handpiece equipped with a disposable tip. The generator produces an electromagnetic field that changes polarity 6 million times per second, causing charged particles to continuously realign within the field. The movement of these particles generates heat due to tissue resistance [6, 9]. High‐frequency energy is selectively delivered to deep skin layers, sparing the epidermis, with a precise cooling system that adjusts the cooling level and duration in five stages to accommodate individual skin temperatures. Growing evidence suggests that the thermal energy delivered to targeted areas promotes the rearrangement and regeneration of the dermal matrix [10, 11]. The heat‐induced denaturation of collagen molecules, followed by collagen contraction and the wound healing response, is believed to support the observed skin tightening and rejuvenation after monopolar RF treatment [10, 12]. Based on these findings, we hypothesized that skin laxity and fine wrinkles would improve following treatment with the Density monopolar RF device.

A review of clinical photography performed by blinded dermatologists in this study reported improvement in skin laxity of the mid and lower face, characterized by tightened jowls. It also diminished nasolabial folds in many subjects, supporting the aforementioned hypothesis. Convergent evidence emerged from analyses of the DermLab Combo‐derived data, which showed statistically significant increases and decreases in VE and RT, respectively, after treatment, suggesting an improvement in skin elasticity. Histological analysis demonstrated an increase in collagen and elastic fiber densities, as well as improved architectural structures after treatment, offering insights into the mechanism responsible for the skin‐tightening effects. These findings suggest that monopolar RF treatment can be considered an effective modality for enhancing skin elasticity. As noted by Tohgasaki et al. [13], collagen VII in the papillary dermis forms a candelabra‐like structure, which, when co‐localized with elastic fibers, appears to prevent wrinkling and sagging. As skin ages, the elongated structure of collagen VII shrinks, and its co‐localization with elastic fibers also diminishes, contributing to the aging of the skin. An EBD (Energy‐based device) targeting this interaction could help counteract these aging‐related changes. Biopsy results from this study indicated a notably increased presence of collagen fibers and elastic fibers in the papillary dermis, likely contributing to enhanced skin elasticity as well as reductions in sagging and fine wrinkles visible in clinical photographs. This study also observed increases in viscoelasticity and decreases in retraction time, although these were not statistically significant. Recently, a study demonstrated that RF (radiofrequency) treatment mitigates age‐related changes at the dermal‐epidermal junctions (DEJs) in animal skin, underscoring its significance [14]. The basement membrane (BM) is essential not only for cell survival and proliferation but also for providing structural support to the DEJ. By regulating various growth factors and regulatory molecules, the BM facilitates critical processes such as keratinocyte adhesion, differentiation, proliferation, and survival. Maintaining BM function is therefore crucial for epidermal regeneration and is considered a fundamental aspect of skin rejuvenation.

Byun et al. [15] demonstrated that RF treatment enhances DEJ protein expression and alleviates structural degradation of the BM. Their research indicated that RF treatment stimulates the release of heat shock proteins (HSP47 and HSA90), TGF‐b, and DEJ proteins, including collagen XVII. Our biopsy findings reveal increased collagen, particularly in the upper papillary dermis where the DEJ is situated.

Fine wrinkle scores were measured using a 3D skin analyzer; however, the results revealed no significant changes pre‐ and post‐treatment. This finding contradicts our hypothesis that the monopolar RF procedure would enhance fine wrinkles, especially considering the histological observations of increased collagen fibers leading to a thicker collagen band in the papillary dermis. This discrepancy between the experimental outcomes and our expectations may be clarified in subsequent studies. The fibrotic band of the DE junction is an observation made in biopsies of fine wrinkles in the cheek region. However, the fine wrinkle assessments captured by 3D include not only those in the entire face but also some slightly coarser wrinkles. We believe this broader inclusion of wrinkle types may explain why the results indicate no significant change. The impact of RF on fine wrinkles has also been addressed in the study by Kim et al. [16].

In addition to its efficacy in enhancing skin elasticity, we hypothesized that monopolar RF treatment might also reduce skin oiliness, thus helping to maintain a proper oil–water balance. Indeed, sebum production, measured by a sebum collecting strip and a strip reading device integrated into the DermLab Combo, significantly decreased following RF treatment in this study. The change in light‐reflecting properties of the strip due to absorption of skin sebum allowed for an objective assessment of skin oiliness. Consistent with this finding, the sebum score, as determined by the 3D skin analyzer, showed substantial declines of 29.60% and 38.90% at 8 and 24 weeks, respectively, post‐treatment compared to baseline readings. Sebum distribution was clearly visualized under the ultraviolet mode, a unique lighting setting of the 3D skin analyzer, from which the sebum score was quantified as the percentage of the area covered by sebum. These results align with previous research on acne patients, indicating that RF treatment suppresses sebum production. A split face study on patients with acne vulgaris using a non‐ablative monopolar RF device (IntraGen, Jeisys Medical Inc., Seoul, Republic of Korea) by Choi et al. demonstrated a 79% reduction in sebumeter readings in the treated group, compared to a mere 2.1% in the untreated group after two treatment sessions [17]. Similarly, 82% of patients with moderate‐to‐severe acne vulgaris reported significant improvement after one or two sessions with a non‐ablative unipolar RF device (ThermaCool TC, Thermage Inc., Hayward, CA, USA), in a clinical study by Ruiz‐Esparza and Barba Gomez [18]. These findings further emphasize the therapeutic potential of thermotherapy, indicating that deep dermal heating may attenuate sebaceous gland activity, promote remodeling of the dermal structure, and decrease sebaceous gland volume. Furthermore, the inhibitory effect of monopolar RF on sebum production appears to be enduring, as evidenced by the sustained significant reduction in sebum score observed 6 months post‐treatment. Considering that genetically ablated sebaceous glands require up to 15 weeks to regenerate in preclinical studies [18], the prolonged effect of RF treatment can plausibly be attributed to the destruction of sebaceous glands by RF‐induced thermolysis. Indeed, previous studies have verified a reduction in sebaceous glands following RF treatment through histological analysis [19, 20].

Considering that the unevenness is more pronounced in oily skin, the statistically significant increase in radiance score in this study, indicating smoothened skin texture, may be attributed to reduced skin oiliness. Interestingly, no significant changes occurred in skin pore scores before and after RF treatment, contradicting the well‐established notion that dilated pores can be improved by a dual mechanism of collagen regeneration and reduced sebum production [21, 22]. Contrary to our findings, Techapichetvanich et al. reported a statistically significant decrease in pore volume by 34% and 38% from the baseline at 1 and 6 months post‐treatment using a non‐ablative monopolar RF device (Thermage FLX, Thermage Inc., Hayward, CA, USA), respectively [23]. These divergent results might reflect variations in inclusion criteria, the number of procedures, and RF‐irradiated areas among the studies. In the previous study, individuals specifically with enlarged pores were given two sessions of RF treatment targeting only areas with enlarged pores. In contrast, in this study, subjects were recruited irrespective of pore condition, and all received a single session of RF treatment adhering to a standard protocol where pulses were delivered to the entire face.

Herein, we conclude that monopolar RF treatment is an effective modality for improving skin laxity and oiliness, based on the quantitative and qualitative data from multiple assessment tools. Histological changes post‐treatment align with earlier findings that monopolar RF treatment facilitates skin rejuvenation through collagen denaturation followed by neocollagenesis [10]. This study has several limitations, including a small sample size, skewed sex distribution of participants, and potential bias from different practitioners. Also, the lack of a control group is another limitation of this study. Further research is needed to evaluate this treatment approach in a larger cohort while controlling for confounding factors to further substantiate our hypothesis.

## Author Contributions

D.H.S. and S.J.L. designed the study. K.Y.S. did a histologic analysis. H.Y. and S.J.C. anaylysed the data. S.J.C. and H.J.R. wrote the paper.

## Ethics Statement

Ethical approval for this study was obtained from the Institutional Review Board of Korea University Ansan Hospital (IRB no. 2023AS0243).

## Consent

Prior to undergoing the procedure, all participants were fully informed of the potential benefits and risks associated with the treatment, and written informed consent was obtained from each patient.

## Conflicts of Interest

The authors declare no conflicts of interest.

## Data Availability

The data that support the findings of this study are available on request from the corresponding author. The data are not publicly available due to privacy or ethical restrictions.
